# Early response evaluation during preoperative chemotherapy for colorectal liver metastases: Combined size and morphology‐based criteria predict pathological response and survival after resection

**DOI:** 10.1002/jso.25796

**Published:** 2019-12-01

**Authors:** Florian Primavesi, Nikolaus Fadinger, Simon Biggel, Eva Braunwarth, Elisabeth Gasser, Susanne Sprung, Georg Göbel, Eva Gassner, Stefan Stättner, Dietmar Öfner

**Affiliations:** ^1^ Department of Visceral, Transplant and Thoracic Surgery Medical University of Innsbruck Innsbruck Austria; ^2^ Department of Radiology Medical University of Innsbruck Innsbruck Austria; ^3^ Institute of Pathology Medical University of Innsbruck Innsbruck Austria; ^4^ Department of Medical Statistics, Informatics and Health Economics Medical University of Innsbruck Innsbruck Austria

**Keywords:** colorectal cancer liver metastases, early tumor shrinkage, morphological criteria, preoperative chemotherapy, response, surgery

## Abstract

**Background:**

Short treatment‐duration with early restaging is crucial to avoid liver injury after preoperative chemotherapy (preopCTX) for colorectal liver metastases (CRLM). Response evaluation according to response evaluation criteria in solid tumors (RECIST) criteria implies several limitations. Early tumor shrinkage (ETS; ≥20% size reduction <6‐12 weeks) or morphological criteria (MC) may better predict oncological outcome.

**Methods:**

In patients undergoing resection after preopCTX between 2003–2017 pathological and radiological response was reassessed according to Blazer classification, ETS, MC, and RECIST within 90 days and correlated with survival.

**Results:**

Seventy‐two patients were included, with a median of two (1‐10) liver lesions, 53% bilobar involvement, and 7% extrahepatic disease. PreopCTX was applied for 3 months in median (1‐6). During restaging after a median of 62 days, presence of ETS was associated with improved median overall survival (OS; 57.1 vs 33.7 months; *P* = .010) and disease‐free survival (16 vs 7.2 months; *P* = .025). MC significantly correlated with major pathological response (*P* = .021). When combining ETS with optimal MC, presence of one or both factors was associated with pathological response (61.5% and 92.3%; *P* = .044) and OS in log‐rank (*P* = .011), and multivariable analysis (hazard ratio [HR] 0.41; 95% confidence interval [CI], 0.19‐0.90 and HR 0.32; 95%CI, 0.11‐0.97).

**Conclusion:**

Response‐grading by combined ETS/MC criteria less than 90 days after preopCTX initiation predicts pathological response and postoperative survival in CRLM.

## 1 INTRODUCTION

The liver represents the most frequent site of metastases in colorectal cancer (CRC), affecting about 15% to 20% of newly diagnosed CRC cases simultaneously,^1^, ^2^ and 20% to 50% of patients in the further course of the disease.^3^ Surgery and ablation remain the major options when aiming for curation.^4^, ^5^, ^6^ Due to utilization of progressive oncosurgical techniques resection rates of metastatic CRC (mCRC) within multimodal concepts currently approach 50% in specialized centers.^1^, ^7^ Hereby, preoperative combination chemotherapy (preopCTX) plays a key role in downsizing advanced colorectal liver metastases (CRLM) to achieve resectability while preserving sufficient future liver volume.^8^, ^9^, ^10^, ^11^, ^12^ Evidence moreover suggests a benefit of preopCTX in a subgroup of patients with primary resectable disease but unfavorable tumor characteristics.^10^, ^13^, ^14^, ^15^ Prolonged preopCTX is associated with considerable toxicity and increases postoperative morbidity and mortality through chemotherapy‐associated liver injury.^16^, ^17^ Hence, restricting preoperative treatment to 3 months or less is recommended by guidelines^10^ to avoid complications, therefore early radiological response assessment is crucial.

Traditionally, response is classified at the time point of maximum tumor reduction according to the response evaluation criteria in solid tumors (RECIST).^18^ Despite advantages in terms of objective standardized assessment, this classification implies several limitations.^16^, ^19^ Besides the prerequisite of inevitable radiological expertize and time‐consuming application, the RECIST stable disease (SD) subgroup is criticized for including both cases with minor tumor shrinkage (0%‐29% size reduction) and minor tumor progression (1%‐19 size increase). Also, patients with complete response (CR) or progressive disease (PD) are rarely represented in typical surgical CRLM cohorts, limiting reasonable risk stratification in clinical practice. To facilitate early restaging, a number of recent trials investigating first‐line CTX in mCRC have evaluated early tumor shrinkage (ETS) as a simple marker for treatment guidance after first restaging. While no consensus definition exists so far, ETS is commonly assessed within 6 to 12 weeks after CTX initiation and defined as a minimum of ≥20% to 30% size reduction in diameters of target lesions.^19^, ^20^, ^21^, ^22^, ^23^, ^24^, ^25^ ETS has been associated with improved progression‐free survival (PFS) and overall survival (OS) in these studies. Since resectability rates in first‐line CTX trials usually range below 15%, and ETS has not yet been validated in a purely surgical cohort, applicability of these results in CRLM patients receiving CTX in a potentially preoperative setting is indeterminate. Furthermore, a previous single center study showed, that response assessment according to morphological criteria (MC) with evaluation of changes in radiological CRLM appearance (tumor density, tumor‐liver border) better predicts histological viability and prognosis after liver resection than size‐based criteria like RECIST.^26^, ^27^ However, response was evaluated after a median of six (up to 24) cycles of CTX, thus transferability to a modern setting of short preoperative treatment with earliest possible restaging remains unclear.

The present study aims to investigate the prognostic value of early response assessment by ETS and MC within 90 days after preopCTX initiation in CRLM patients undergoing liver resection.

## 2 MATERIALS AND METHODS

### 2.1 Patient cohort and data

**Figure 1:**
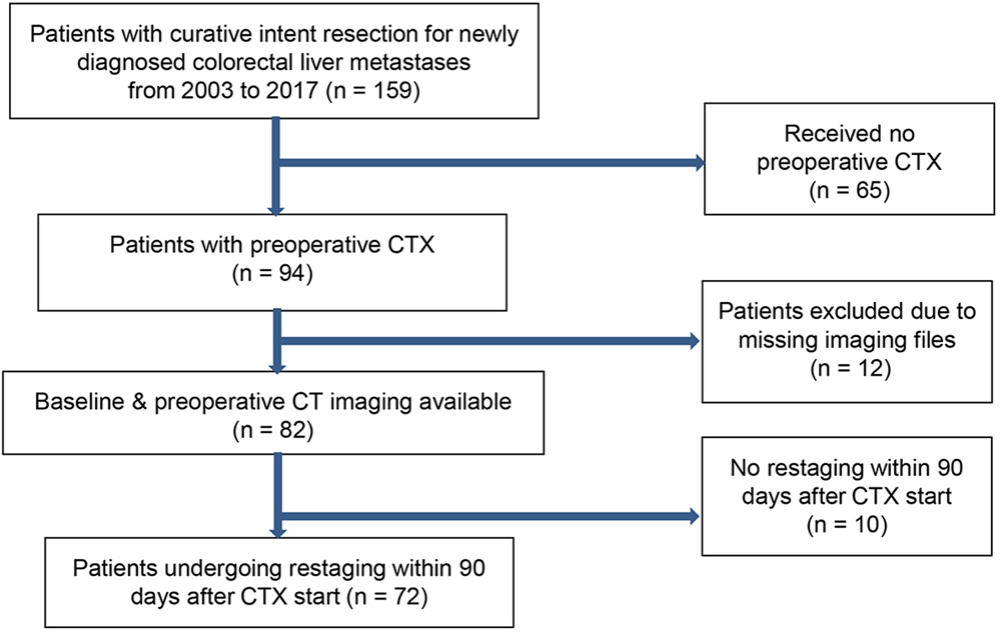
CONSORT flow chart of patient selection for this study. CTX, chemotherapy [Color figure can be viewed at wileyonlinelibrary.com]

Patients undergoing curative‐intent liver resection for newly diagnosed CRLM at Medical University of Innsbruck between 2003 and 2017 were reviewed from our database (Figure 1). The study was approved by the local ethics committee (number: 1033/2017) waiving the need for individual informed consent. Enrolled patients had synchronous or metachronous (≥6 months after CRC) CRLM without prior surgical or locoregional metastases treatment. Cases without preopCTX or available imaging within 90 days after treatment initiation were excluded. Major resection comprised ≥3 liver segments or ≥6 atypical resections/ablations, postoperative 90‐day‐morbidity was graded according to the Dindo‐Clavien classification.^28^, ^29^ OS was defined as time from metastasectomy to death or to last follow‐up with known alive status, derived from patient records and cross‐checked with national survival data.^30^ Disease‐free survival (DFS) was defined as time to occurrence of any relapse or to last follow‐up date of known tumor‐free status.

### 2.2 Radiological assessment

**Figure 2:**
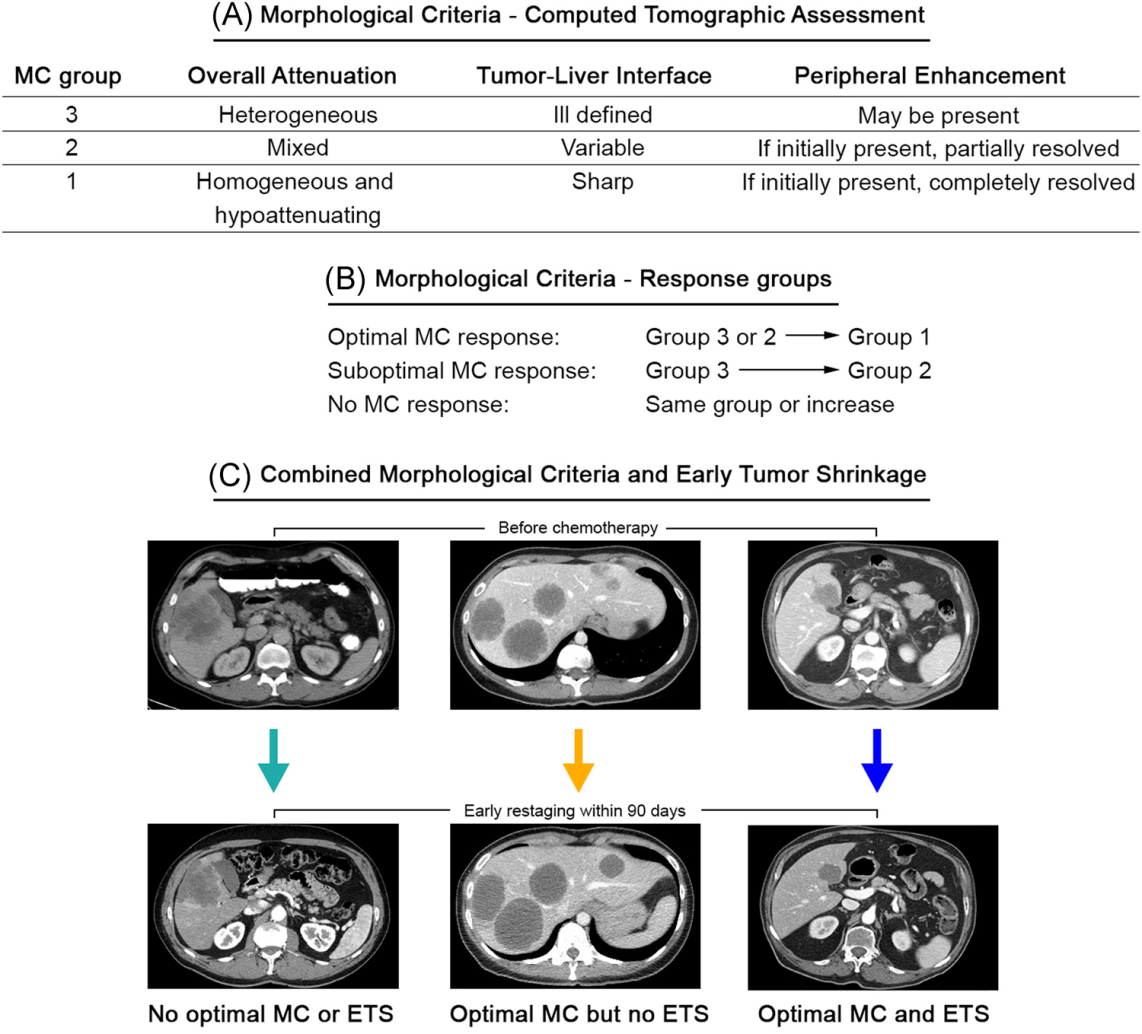
Grading according to the morphological criteria (MC) classification A, Liver target lesions are grouped at baseline and during restaging according to the MC. B, Response is evaluated according to the change between groups. C, Combination of optimal MC (lesions with homogeneous attenuation and sharp tumor‐liver interface) and early tumor shrinkage (ETS; ≥20% size reduction of target lesions). Left: during restaging, neither optimal MC response nor ETS is present. Middle: optimal MC but no sufficient size reduction (<20%). Right: both optimal MC and size reduction ≥20% are present [Color figure can be viewed at wileyonlinelibrary.com]

Two radiologists with >30 years combined clinical experience blinded for treatment and outcome of patients reviewed baseline and first restaging CT images for response according to three previously reported criteria: (a) RECIST 1.1.^18^; (b) ETS (≥20% shrinkage within 90 days after CTX start)^19^; (c) MC^26^ (3 groups; Figure 2A,B). Regarding MC assessment, the preopCTX target lesion appearance was graded in terms of overall attenuation (heterogeneous, mixed, or homogeneous), tumor‐liver interface (ill‐defined, variable or sharp border), and peripheral enhancement (present vs not present; Figure 2A). Accordingly, metastases were allocated to MC group 3 (heterogeneous with ill‐defined border) or MC group 1 (homogeneous with sharp border) or MC group 2 (mixed appearance). During restaging, the same lesions were again assessed according to these criteria and response was rated as optimal MC response when previous MC group 3 or 2 lesions had changed to group 1, suboptimal MC response when MC group 3 lesions had changed to group 2 or no MC response when the restaging group was identical or even increased (Figure 2B).

Computed tomography were performed with intravenous contrast media evaluated in arterial phase with slice thickness ≤5 mm. A maximum two target liver lesions and one additional lesion per organ in case of extrahepatic disease was assessed. In cases with mixed response, the lesion with worst response was recorded for overall grading. For diverging gradings recorded by the two radiologists, consensus was achieved through individual case discussion. RECIST groups included CR, partial response (PR), SD, or PD. ETS was divided into presence or absence of ETS. Combination of ETS and optimal MC was applied as exemplarily shown in Figure 2C.

### 2.3 Pathological response

All surgical specimens were re‐evaluated by an expert oncopathologist (S.S.) for extent of response according to the classification described by Blazer.^31^ Briefly, this assesses the percentage of viable tumor cells and grades into three groups: CR (no viable tumor cells), major response (1%‐49% viability) and minor response (≥50% viability).

### 2.4 Statistical analysis

Nominal variables were reported as frequencies/percentages, continuous variables as medians with standard deviation or range. Differences between groups were analyzed by *χ* ^2^ or Fisher's exact test. Correlation between nominal variables was assessed with Cramer's *V*, whereby a level of more than 0.250 was considered indicating a strong relationship. Kaplan‐Meier survival curve estimations were plotted with the log‐rank test for subgroup comparison. Cox‐proportional hazards regression analysis (enter method) was performed to assess associations of clinical factors with OS. All calculations were performed with SPSS Version 21 (IBM Inc, Armonk, NY), a two‐tailed *P* value of <.05 was considered significant.

## 3 RESULTS

Between 2003 and 2017 159 patients underwent curative‐intent liver resection for newly diagnosed CRLM, of which 72 were assessed in the final cohort according to the study inclusion criteria (Figure 1). Details of patient, tumor, and treatment characteristics are depicted in Table 1. While our cohort represents typical western world CRLM patients, notable the majority had synchronous disease with advanced, multiple liver metastases up to 10.5 cm, preoperatively treated mostly with oxaliplatin‐based chemotherapy and additional biologicals. 90‐day‐morbidity occurred in 23 patients (31.9%), whereby 9 (12.5%) experienced severe complications (≥3b Dindo‐Clavien) and one patient (1.4%) deceased. After a median follow‐up of 35.4 months (SD 37.3), the estimated median OS and DFS was 48.4 months (95% confidence interval [CI], 35.2‐61.6) and 11.0 months (95%CI, 6.8‐15.3), respectively.

**Table 1:** Patient, tumor, and treatment characteristics in the final cohort (n = 72)

Variable	Number (%)
Patient	
Male sex	43 (59.7%)
Age (median; SD)	59.2 (11.1)
BMI (median; SD)	24.8 (4.2)
ASA score (median; SD)	2 (0.59)
Primary tumor	
Location (sidedness)	
Right sided (cecum to transverse colon)	16 (22.2%)
Left sided (splenic flexure to sigmoid)	28 (38.9%)
Rectum	28 (38.9%)
Nodal status	
Positive	46 (63.9%)
Negative	26 (36.1%)
Not available	
Metastases	
Synchronous timing	60 (83.3%)
Whole‐RAS (KRAS/NRAS) mutation status	
Wild‐type	36 (50%)
Mutant	19 (26.4%)
Not available	17 (23.6%)
Size of largest liver lesion (median; range)	21.5 mm (5‐105)
Number of liver lesions (median; range)	2.0 (1‐10)
Bilobar involvement	38 (52.8%)
Extrahepatic disease present	5 (6.9%)
Treatment	
Duration of CTX (in months: median; range)	3.0 (1‐6)
Type of CTX (missing=1)	
5‐FU based	3 (4.2%)
Oxaliplatin‐based dual	50 (70.4%)
Irinotecan based dual	13 (18.3%)
Oxaliplatin/irinotecan based triple	5 (7%)
Additional biologicals (missing=1)	
EGFR antibody added	23 (32.4%)
VEGF antibody added	21 (29.6%)
Type of liver resection	
Major resection	31 (43.1%)
Anatomical	25 (34.7%)
Nonanatomical	16 (22.2%)
Both anatomical and nonanatomical	31 (43.1%)

Abbreviations: 5‐FU, 5‐fluorouracil; ASA, American society of anesthesiologists; BMI, body mass index; CTX, chemotherapy; EGFR, epidermal growth factor receptor; KRAS, kirsten rat sarcoma viral oncogene homolog; mm, millimeter; NRAS, neuroblastoma RAS viral oncogene homolog; SD, standard deviation; VEGF, vascular endothelial growth factor.

### 3.1 Radiological and pathological response

The median time from CTX start to first restaging was 62 days (SD 16.5). Results of radiological assessment at first restaging according to the different classifications are reported in Table 2. ETS defined by ≥20% tumor shrinkage was present in 70.8%, suboptimal or optimal MC response in 19.4% and 26.4%, respectively. Evaluation according to the RECIST criteria resulted in only few patients classified as PD (n = 2; 2.8%) or CR (n = 3; 4.2%) compared to 37.5% and 55.6% SD or PR cases, respectively. Neither the type of CTX nor additional biologicals were associated with presence of ETS or optimal MC response MC (all *P* > .1). Pathological response was determinable in 68 patients (5.6% missing) with grading according to Blazer as follows: minor response n = 24 (35.3%), major response n = 39 (57.4%), CR n = 5 (6.9%).

**Table 2:** Radiological response according to the three different classifications

Response classification	Number (percentage) of patients according to subgroup within classification			
Early tumor shrinkage (ETS)	No (<20% size reduction)	Yes (≥20%)		
	21 (29.2%)	51 (70.8%)		
Morphological criteria (MC)	No response	Suboptimal response	Optimal response	
	39 (54.2%)	14 (19.4%)	19 (26.4%)	
RECIST 1.1 criteria	Progressive disease (PD)	Stable disease (SD)	Partial response (PR)	Complete response (CR)
	2 (2.8%)	27 (37.5%)	40 (55.6%)	3 (4.2%)

Abbreviation: RECIST, response evaluation criteria in solid tumors.

### 3.2 OS according to radiological and pathological response

**Figure 3:**
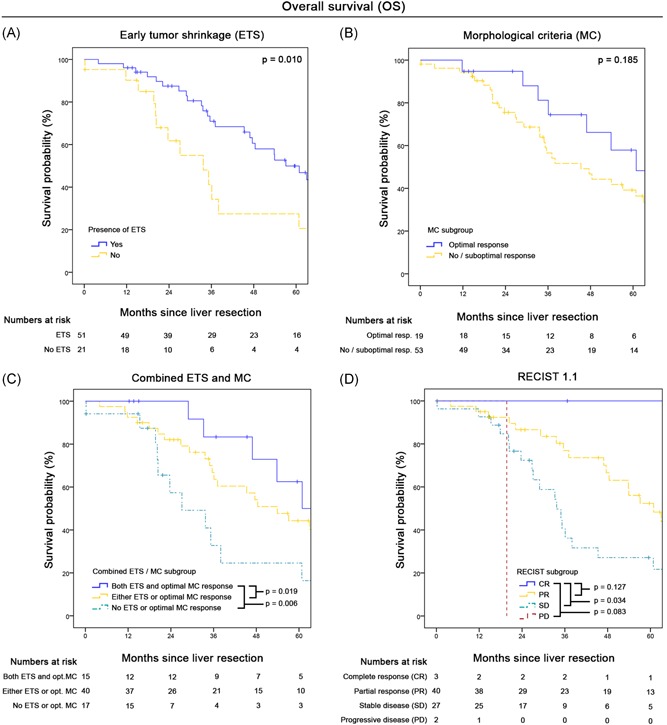
A‐D, Overall survival according to different radiological classification systems: A, Presence or absence of early tumor shrinkage (ETS ≥20%). B, Optimal vs suboptimal or no morphological response. C, Combination of ETS and MC. D, RECIST 1.1 criteria. MC, morphological criteria; RECIST, response evaluation criteria in solid tumors [Color figure can be viewed at wileyonlinelibrary.com]

Figure 3 *A‐D* shows Kaplan‐Meier OS curve estimates according to ETS, MC, combined ETS/MC, and RECIST 1.1 classifications. ETS‐classification provided significant discrimination between patients with presence (n = 51) and absence (n = 21) of ≥20% shrinkage within 90 days after CTX start: median OS 57.1 months (95%CI, 45.1‐69.1) vs 33.7 months (95%CI, 20.1‐47.3; *P* = .010). According to MC, patients with optimal response (n = 19) had a median OS of 60.9 months (95%CI, 15.0‐106.8) vs 33.6 months (95%CI, 0.0‐72.4) in cases with suboptimal response (n = 14) and 45.3 months (95%CI, 30.5‐60.1) in those without response (n = 39; overall *P* = .412). When patients were grouped according to optimal MC response vs suboptimal/no MC response the median OS was 60.9 months (95%CI, 15.0‐106.8) vs 45.3 months (95%CI, 29.8‐60.8; *P* = .185).

To evaluate the value of combined size and morphology‐based response assessment, we further consolidated both the ETS and grouped‐MC criteria. As depicted in Figure 3C, this resulted in three subgroups of patients with reasonable case numbers and significantly different outcome. The median OS was 60.9 months (95%CI, 20.0‐101.8) in patients with both ETS and optimal MC (n = 15), compared to 53.9 months (95%CI, 38.4‐69.4; *P* = .019) in cases with only either ETS or optimal MC (n = 40) and 27.1 months (95%CI, 10.6‐43.6; *P* = .006) without any of the two criteria (n = 17; overall *P* = .011). The results remained statistically significant, when excluding the one case with 90‐day postoperative mortality (overall *P* = .025).

Graded by RECIST, no patient with CR (n = 3) died during follow‐up (median OS not computable), compared to a median OS of 60.9 months (95%CI, 47.3‐74.5) in PR patients (n = 40; *P* = .127), 33.7 months (95%CI 2466‐41.0) in SD (n = 27; *P* = .034) and 19.7 months (95%CI not computable) in PD patients (n = 2; *P* = .083).

Patients with complete pathological response showed a 5years‐OS of 53.3% (median not reached) compared to 44.8% in cases with major response (median 53.8 months; 95%CI, 42.0‐65.6; *P* = .304) and 39.1% after minor response (median 45.3 months; 95%CI, 28.9‐61.7; *P* = .203; overall *P* = .440). Also, significance was not reached when grouping major and minor response (median 47.6 months; 95%CI, 31.1‐64.1; *P* = .244) or complete and major response (median 53.9 months; 95%CI, 36.7‐71.1; *P* = .429).

### 3.3 DFS according to radiological and pathological response

**Figure 4:**
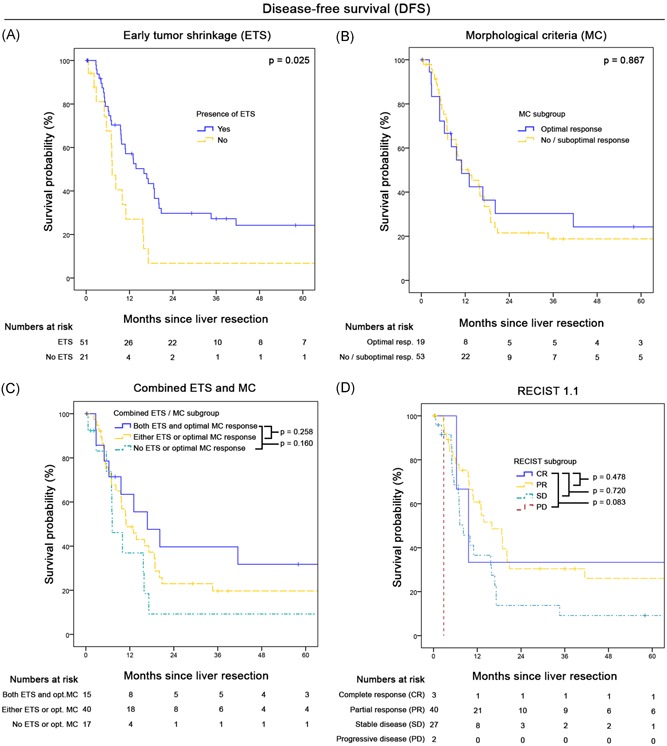
A‐D, Disease‐free survival according to different radiological classification systems: A, Presence or absence of early tumor shrinkage (ETS ≥20%). B, Optimal vs suboptimal or no morphological response. C, Combination of ETS and MC. D, RECIST 1.1 criteria. MC, morphological criteria; RECIST, response evaluation criteria in solid tumors [Color figure can be viewed at wileyonlinelibrary.com]

During follow‐up 50 patients (69.4%) experienced recurrence, resulting in an estimated 5‐year DFS of 20%. Figures 4A‐D provides DFS curves according to radiological response. Only the presence of ETS was significantly associated with DFS: median 16 months (95%CI, 9.3‐22.7) vs 7.2 months (95%CI, 5.7‐8.7; *P* = .025). Response according to MC was not significantly associated with DFS (*P* = .834). The combination of ETS and MC resulted in a median DFS of 16.8 months (95%CI, 4.7‐28.9) when both factors were present compared to 11 months (95%CI, 6.4‐15.7) and 7.2 months (95%CI, 2.6‐11.8) in cases with only one or none factor present (*P* = 0.318). Also, RECIST criteria did not result in clinically practical DFS curve discrimination with a median DFS in the PD group of 2.8 months (95%CI not computable), 8.2 months in SD patients (95%CI, 4.8‐11.6), 16 months in PR cases (95%CI, 10.6‐21.4), and 9.6 months in CR patients (95%CI, 4.3‐14.9). Pathological response did not significantly predict DFS (minor response median DFS: 9.8 months (95%CI, 7.8‐11.8) vs major/CR 15.7 months (95%CI, 11.4‐20.0; *P* = .376).

### 3.4 Correlation of radiological and pathological response

**Figure 5:**
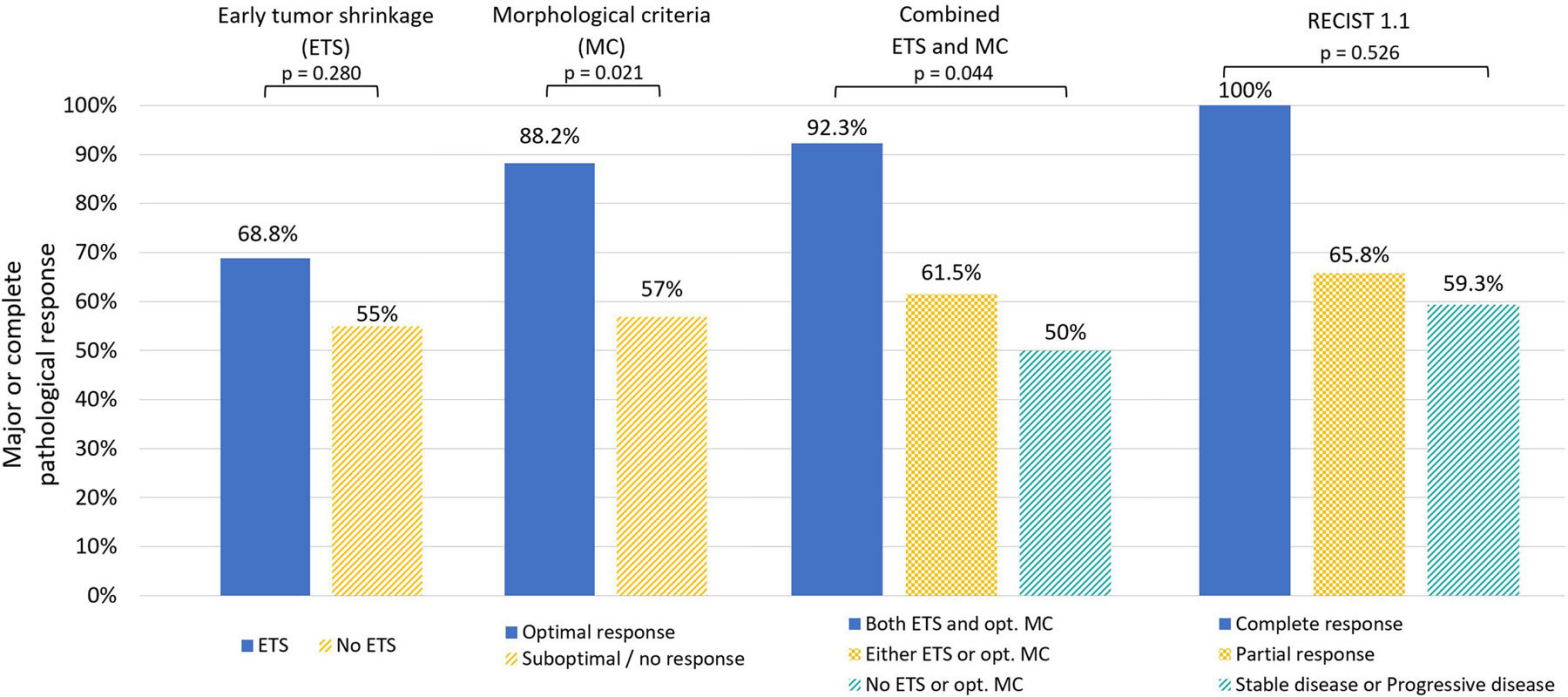
Rate of major or complete pathological response according to different radiological response criteria. ETS, early tumor shrinkage; MC, morphological criteria; RECIST, response evaluation criteria in solid tumors [Color figure can be viewed at wileyonlinelibrary.com]

Analyzing the association between radiological and pathological response (Figure 5), we found a strong correlation between MC criteria and pathological response according to the Blazer classification: 88.2% of patients with optimal MC response showed a major or complete pathological response compared to 57% in those with no or suboptimal MC response (*P* = .021; Cramer's *V *= 0.284). Presence or absence of ETS was not significantly correlating with major or complete pathological response (68.8% vs 55%; *P* = .280; Cramer's *V *= 0.131). Intriguingly, the combined approach of both ETS and optimal MC stratification revealed a very strong correlation with increasing rates of pathological response in each subgroup. While patients without ETS or optimal MC experienced major or CR in only 50% of cases, the rate was 61.5% with one radiological factor present and 92.3% with both imaging criteria present (*P* = .044; Cramer's *V *= 0.298). In contrast, radiological response according to RECIST criteria showed only a weak, nonsignificant correlation with major or complete pathological response: 100% in RECIST‐CR patients, compared to 65.8% in partial response and 59.3% in stable or PD patients (*P* = .526; Cramer's *V *= 0.172).

### 3.5 Multivariable analysis of factors associated with OS

Table 3 shows association of different patient‐related factors, tumor characteristics, and treatment variables with OS assessed by Cox‐proportional hazards regression. In univariable analysis the presence of ETS and/or optimal MC after restaging within 90 days was significantly associated with improved OS (hazard ratio [HR] 0.41 and 0.28; *P* = .016 and *P* = .012, respectively). In contrast, nodal positivity and RAS mutation of the primary tumor (both HR 2.24; *P* = .035) were linked to worse OS, as was the need for major liver resection (HR 1.99; *P* = .034). All of these factors except nodal positivity remained statistically significant in the multivariable regression model.

**Table 3:** Univariable and multivariable cox‐regression analysis of factors associated with overall survival

	Univariable analysis		Multivariable analysis	
Variable	HR (95%CI)	*P*	HR (95%CI)	*P*
Patient				
Male sex	0.94 (0.50‐1.78)	.844		
Age ≥60 y	0.92 (0.49‐1.72)	.783		
BMI ≥25	0.51 (0.25‐1.03)	.062		
Primary tumor				
Right sided location	2.00 (0.94‐4.26)	.072		
Nodal positivity	2.24 (1.06‐4.73)	.035	1.65 (0.72‐3.79)	.235
RAS mutational status				
Wild‐type	Ref.		Ref.	
Mutated	2.24 (1.06‐4.74)	.035	2.25 (1.02‐4.98)	.046
Unknown	1.08 (0.48‐2.41)	.853	1.38 (0.60‐3.18)	.451
Metastases				
Synchronous timing	0.98 (0.45‐2.30)	.976		
Size of largest liver lesion ≥20 mm	0.95 (0.51‐1.79)	.884		
Number of liver lesions ≥ 2	0.94 (0.49‐1.80)	.843		
Bilobar involvement	1.42 (0.74‐2.70)	.288		
Extrahepatic disease present	2.01 (0.61‐6.66)	.252		
CTX response				
Radiological response: ETS and/or optimal MC				
None present	Ref.		Ref.	
One factor present	0.41 (0.20‐0.84)	.016	0.41 (0.19‐0.90)	.026
Both present	0.28 (0.10‐0.75)	.012	0.32 (0.11‐0.97)	.044
Pathological response (Blazer)				
Minor response	Ref.			
Major response	0.83 (0.42‐1.67)	.609		
Complete response	0.39 (0.09‐1.73)	.216		
Treatment				
Preoperative CTX ≥ 3 mo	1.24 (0.62‐2.46)	.548		
Combination CTX (compared to 5‐FU)	1.03 (0.31‐3.36)	.964		
Additional biological given	1.06 (0.55‐2.04)	.852		
Major liver resection	1.99 (1.05‐3.76)	.034	2.29 (1.14‐4.61)	.020

Abbreviations: 5‐FU, 5‐fluorouracil; BMI, body mass index; CI, confidence interval; CTX, chemotherapy; ETS, early tumor shrinkage; HR, hazards ratio; MC, morphological criteria; RAS, rat sarcoma viral oncogene homolog.

## 4 DISCUSSION

This study analyzed the prognostic value of different radiological response criteria early during preopCTX in 72 CRLM patients. OS was validated through official national data and the study specific workup ensured meticulous reassessment of all preoperative CT images and postoperative specimens by trained radiologists and histopathologists blinded to oncological outcomes. We first determined, that early imaging assessment within 90 days according to ETS provides significant risk stratification regarding OS and DFS after liver surgery. ETS has previously already been established as a valuable factor to assess early response and predict PFS and OS in oncological first‐line CTX trials.^19^ However, in these studies, only a limited number of patients finally underwent liver resection and no specific surgical cohort sub‐analysis has been published.

Moreover, our results support findings of others, that morphology‐based criteria such as the MC are more precisely correlating with pathological response than size‐based criteria such as RECIST or ETS.^26^, ^27^ Interestingly though, in our cohort MC response was not significantly associated with OS, which is presumably owed to the limited number of patients experiencing optimal MC response, since previous studies have also only shown a clear significance when larger cohorts (>​​​​​200 patients) were evaluated.^26^, ^27^ Over the last years many dedicated hepatobiliary surgeons involved in oncosurgical treatment of advanced CRLM have questioned the value of purely size‐based response criteria such as RECIST to correctly identify patients who will benefit from resection after preopCTX. Intriguingly, to the best of our knowledge, the present study indeed for the first time provides data suggesting that combined application of both size‐based as well as morphology‐based criteria may more precisely stratify for response on a cellular level and consecutively expected postoperative survival. In our analysis, a combination of ETS and MC after a median of 62 days strongly predicted pathological response and survival. In patients with none of the two response criteria present, substantial pathological response is only present in every second patients and long‐term survival is poor, with estimated 5‐years OS rates of <20% and 5‐year DFS <10%. In contrast, cases with both ETS and optimal MC response experience major or complete pathological response in a striking 92%, resulting in 5‐year OS and DFS of more than 60% and 30%, respectively. Furthermore, multivariable analysis has confirmed the independent association of combined ETS/MC criteria with OS after resection even when corrected for other established risk factors such as nodal positivity and RAS mutation of the primary tumor or necessity of major liver resections.

Importantly, the combined ETS/MC criteria are easy to apply in clinical practice. Evaluation of maximally two target liver lesions concerning a shrinkage of 20% of their total diameter and the presence of homogenous and hypoattenuating appearance with a well‐defined, sharp tumor‐liver interface at restaging requires little radiological expertize and time. Despite the rather subjective approach applied during MC grading, good interobserver agreement between different radiologists was previously demonstrated, ensuring appropriate validity.^27^ In contrast, assessment by RECIST criteria involves several clinical limitations to be acknowledged.^16^, ^18^ RECIST grading is time‐consuming and results in four subgroups of which one is criticized for its' cut‐off ranges (SD) and two others are of limited clinical usefulness due to their rare frequency (CR and PD) in modern neoadjuvant CRLM treatment. Furthermore, it has been previously demonstrated, that RECIST is less precise than MC in predicting pathological response and OS in patients receiving bevacizumab‐containing preopCTX for CRLM.^27^

The limitations of this study primarily include its retrospective nature and potential inclusion bias. A prospective trial including all patients undergoing chemotherapy for advanced but potentially ever resectable CRLM would provide a more complete picture in this challenging cohort. Currently ongoing large‐scale projects on complex CRLM surgery such as the EORTC‐ESSO 1409/CLIMB registry^32^ will hopefully also provide insights in these specific issues. Although first‐line chemotherapy trials for mCRC have already established ETS as a prognostic marker, the low resection rates in these studies do not reflect the typical surgical cohort of specialized hepatobiliary surgeons. We therefore aimed to address the following clinical scenario: In patients with potentially resectable, borderline‐resectable or primary unresectable CRLM deemed to undergo preopCTX, which classification should be used by hepatobiliary surgeons to assess radiological response early within 2 to 3 months of initiation of chemotherapy to predict pathological response and postoperative prognosis? In this regard, our study represents the first of its kind, with statistically sufficient sample size and a methodology that attempts to limit the restrictions of its' retrospective design. However, applicability of the combined ETS/MC classification in other centers or geographical regions (eg Asia) or in patients undergoing modern triple CTX (eg FOLFOXIRI^8^, ^33^; only 7% in our cohort) justifies further investigation. Therefore, prospective, international validation, ideally with centralized radiological and pathological assessment would be desirable in the future.

## 5 CONCLUSIONS

This study presents a novel combination of established imaging criteria to assess early response during short‐term preopCTX for advanced CRLM. The combined ETS/MC classification correlates with pathological response and OS, facilitates practical clinical applicability and fosters risk stratification to guide treatment by liver surgeons.

## SYNOPSIS

During preoperative chemotherapy for colorectal cancer liver metastases, routinely the response evaluation criteria in solid tumors (RECIST) criteria are applied to assess radiological response. This study examines a defined set of surgical patients with liver resection after short‐term chemotherapy and shows, that a combination of a simple size‐based response criterion (≥20% shrinkage within 6‐12 weeks) and morphological criteria significantly predicts pathological response and postoperative survival. Easy applicability in clinical practice compared to the RECIST classification allows for effective preoperative risk stratification.
